# Association of waist-calf circumference ratio, waist circumference, calf circumference, and body mass index with all-cause and cause-specific mortality in older adults: a cohort study

**DOI:** 10.1186/s12889-023-16711-7

**Published:** 2023-09-12

**Authors:** Miao Dai, Bin Xia, Jiangqi Xu, Weiyun Zhao, Dongdong Chen, Xiang Wang

**Affiliations:** 1 Department of Geriatrics, Jiujiang First People’s Hospital, Jiujiang, 332000 Jiangxi China; 2 Department of Cardiology, Jiujiang First People’s Hospital, Jiujiang, 332000 Jiangxi China

**Keywords:** Waist-calf circumference ratio, Waist circumference, Calf circumference, Mortality, Older adults

## Abstract

**Background:**

Waist circumference (WC), calf circumference (CC), and body mass index (BMI) have been independently linked to mortality. However, it's not yet clear how the waist-calf circumference ratio (WCR) relates to mortality. This study aims to investigate the relationship between WCR, WC, CC, and BMI with all-cause and cause-specific mortality in older adults.

**Methods:**

In the 2014 Chinese Longitudinal Healthy Longevity Survey, 4627 participants aged 65 years and older were included, and they were subsequently followed up in 2018. Cox proportional hazards models were utilized to estimate hazard ratios (HRs) and 95% confidence intervals (CIs) for all-cause and cause-specific mortality, based on WCR, WC, CC, and BMI.

**Results:**

During a median follow-up of 3.4 years, 1671 deaths (36.1%) occurred. Compared to the second quartile of WCR, the highest quartile had a higher risk of mortality from all causes (HR 1.42, 95%CI 1.24–1.64), cardiovascular disease (CVD) (HR 1.88, 95%CI 1.38–2.56), and other causes (HR 1.37, 95%CI 1.15–1.63). The first and fourth quartiles of WC had HRs of 2.19 (1.00–4.79) and 2.69 (1.23–5.89), respectively, for cancer mortality. The highest quartile of CC was associated with a lower risk of all-cause and other-cause mortality, whereas the lowest quartile was associated with a higher risk of all-cause, CVD, and other-cause mortality compared to the second CC quartile. Additionally, the lowest quartile of BMI was associated with a higher risk of all-cause and respiratory disease mortality. Interaction analyses showed that the effects of CC on all-cause and CVD mortality were more pronounced in adults aged ≥ 80 years (P-interaction < .05).

**Conclusions:**

Higher WCR and lower CC increased the risk of all-cause, CVD, and other-cause mortality. Lower BMI was associated with higher all-cause and respiratory disease mortality risk, while WC only predicted cancer mortality.

**Supplementary Information:**

The online version contains supplementary material available at 10.1186/s12889-023-16711-7.

## Background

Obesity refers to an excessive accumulation of body fat, often characterized by a body mass index (BMI) above a defined threshold [1]. Central obesity, on the other hand, specifically involves the accumulation of fat around the abdominal area, measured by waist circumference (WC) [2]. While obesity as a whole is linked to a heightened risk of chronic diseases, central obesity holds distinct importance. Research suggests that central obesity, due to its impact on visceral fat surrounding vital organs, was more strongly associated with chronic disease risk (including cardiovascular diseases, type 2 diabetes, metabolic syndrome, and certain cancers) [3–5] and mortality [6, 7], than general obesity. This distinction highlights the importance of considering both overall obesity and central obesity as distinct factors contributing to chronic disease and mortality susceptibility. Given the increasing prevalence of obesity and the associated health dangers, accurately and reliably establishing methods to measure central adiposity becomes crucial in addressing the growing public health burden linked to obesity-related health issues.

BMI is a commonly used measure of obesity and has been widely used as a predictor of mortality [3, 8, 9]. However, BMI does not differentiate between fat and muscle mass and does not account for body fat distribution, which is an important predictor of mortality [10]. WC is a practical and straightforward tool for assessing abdominal obesity and is strongly associated with CVD [11] and mortality [12]. However, in older people, WC alone may not reflect body composition accurately, as they are more likely to have a higher prevalence of central obesity and lower muscle mass than younger individuals [3]. In contrast, calf circumference (CC) is a marker of muscle mass and has been associated with lower mortality risk [13]. Recently, the waist-calf circumference ratio (WCR) has been proposed as a potential measure of body composition as it combines measures of central obesity and muscle mass into a single metric. WCR was associated with carotid atherosclerosis [14], cognitive impairment [15], and health-related quality of life [16] and has been suggested as a better predictor of health outcomes than BMI, WC, or CC alone [15]. However, to the best of our knowledge, no studies have investigated the association between WCR and mortality risk among older adults. Additionally, evidence regarding the association between CC and cause-specific mortality in older adults is limited.

Therefore, our study aims to investigate the association of anthropometric measures, including WCR, WC, CC, and BMI, with all-cause and cause-specific mortality among older people in a community-based cohort study.

## Methods

### Study design and participants

The Chinese Longitudinal Healthy Longevity Survey (CLHLS) is an ongoing prospective cohort study being conducted in 22 of China's 31 provinces aimed at investigating factors affecting the health and longevity of Chinese older adults. After launching in 1998, the CLHLS conducted follow-up interviews in 2000, 2002, 2005, 2008, 2011, 2014, and 2018, with responses greater than 90%. During each follow-up, new participants are enrolled to reduce attrition due to death and loss of follow-up. Individuals who were still alive were re-interviewed every two to four years. Family members of deceased individuals were asked about the cause and date of death, health service usage, and health status before death. There is an in-depth description of CLHLS elsewhere [17].

The baseline data for this study was from the 2014 CLHLS, because there were many missing values in the CC in the previous waves, and only the 2018 collected data on cause-specific deaths. The follow-up was conducted in 2018. Among 7192 older adults, we excluded participants who were younger than 65 years old (*n* = 85), were lost to follow-up in 2018 (*n* = 1511), had an incorrect follow-up date (*n* = 6), had missing data or incorrect data on CC or WC (*n* = 605), and had missing data on height or weight (*n* = 358). Finally, a total of 4627 participants were included to analyze the association of WCR, WC, CC, and BMI with all-cause and cause-specific mortality in older adults. Supplementary Fig. 1 shows a detailed description of the inclusion and exclusion process.

### Anthropometric assessment

Standard operating procedures were employed to measure WC, CC, height, and weight. To ensure accuracy, the measuring tape was placed flat on the skin, avoiding any compression during the measurement of WC and CC. The midpoint between the lower ribs and the iliac crest at the end of normal expiration was selected as the measurement point for WC, whereas CC was measured at the point of maximum calf circumference. Height was assessed using a portable stadiometer while the participants were barefooted, and weight was measured on a weight scale with light indoor clothing and no shoes. The WCR was determined as the ratio of WC (in centimeters) and CC (in centimeters). Additionally, the BMI was computed as the quotient of weight (in kilograms) and the square of height (in meters).

### Outcome assessment

The classification of causes of mortality was conducted by referring to the codes of the international statistical classification of Diseases, 10th revision (ICD-10). The primary outcomes were mortality from all causes, as well as specific causes such as CVD (codes I00-I99), respiratory diseases (codes J00–99), cancer (codes C00-C97), and other causes. The next of kin of participants provided information on reported deaths. Survival time was calculated from the first interview date until either the date of death or the last follow-up.

### Covariates assessment

The structured questionnaire was used to obtain covariates in this study. The questionnaire was divided into two sections, namely sociodemographic and health characteristics. The sociodemographic section consisted of age, sex (male or female), education (0 years, 1 year or more), type of residence (rural area or urban area), and marital status (married or others—divorced, widowed, or never married). The health characteristics section covered various aspects such as smoking habits (never, current or former), alcohol consumption (never, current or former), regular exercise routine (never, current or former), intake of vegetables (never, occasionally, or almost daily), intake of fruit (never, occasionally, or almost daily), intake of meat (never, occasionally, or almost daily), intake of fish (never, occasionally, or almost daily), and self-reported diseases diagnosed by a doctor, which included hypertension (yes or no), heart disease (yes or no), diabetes mellitus (yes or no), cerebrovascular disease (yes or no), respiratory disease (including pneumonia, bronchitis, emphysema, and asthma) (yes or no), and cancer (yes or no).

### Statistical analysis

We presented baseline characteristics as means and standard deviations (SDs) for continuous variables and as percentages for categorical variables. Detailed information on the number of missing variables is shown in Supplementary Table 1. In handling missing values, we employed the Multiple Imputations by Chained Equations method with predicted mean matching, performing five imputations to enhance the robustness and reliability of our analysis [18]. The predicted mean matching guarantees that the imputed values adhere to a suitable range by aligning the predicted value with the nearest data point within the dataset.

We selected the second quartile of WCR, WC, CC, and BMI as the reference group, and utilized Cox proportional hazards models to estimate hazard ratios (HRs) and 95% confidence intervals (95% CIs) for all-cause and cause-specific mortality associated with the remaining quartile groups. We utilized Kaplan–Meier survival analysis to construct survival curves for anthropometric measures and assessed differences between groups using log-rank testing. No potential violations of the proportional hazard assumption were detected based on Schoenfeld's residuals. Multivariate models were adjusted for various baseline factors, including age, sex, residence, education, marital status, smoking status, drinking status, regular exercise, intake of vegetables, intake of fruit, intake of meat, intake of fish, BMI, hypertension, diabetes mellitus, cerebrovascular disease, heart disease, respiratory disease, and cancer. To investigate the independent impact of anthropometric measures on mortality, we adjusted for WC when evaluating CC, and vice versa, and adjusted for WC and CC when evaluating BMI. The crude incidence rate (IR) (per 1000 person-years) of all-cause and cause-specific mortality was estimated. We conducted a restricted cubic spline analysis, with four knots at the 5th, 35th, 65th, and 95th percentiles of changes in the distribution of WCR, WC, CC, and BMI to investigate the dose–response relationship between changes in these factors and the risk of mortality. The reference value was set as the 50th percentile of WCR (2.67), WC (80.00 cm), CC (30.00 cm), and BMI (21.48 kg/m^2^). We tested interaction terms of potential covariates (age and sex) with WCR, WC, CC, and BMI on mortality outcomes and performed subgroup analyses.

To ensure the validity and reliability of our research outcomes, we performed sensitivity analyses. Firstly, we conducted complete case analyses after excluding participants with incomplete covariate data. Secondly, we excluded individuals with prevalent major chronic diseases, including heart disease, diabetes mellitus, cerebrovascular disease, and cancer at baseline, to explore possible reverse causality.

All statistical analyses were performed using R statistical software version 4.1.3 (R Foundation for Statistical Computing), and the significance threshold was a two-tailed *P* value < 0.05.

## Results

### Basic characteristics of participants

Among the 4627 participants, 52.3% were female, with a mean (SD) age of 84.7 (10.2) years. During a median follow-up duration of 3.5 years (14815.1 person-years), a total of 1671 (36.1%) deaths were identified. 382 (22.9%) deaths were from CVD, 174 (10.4%) from respiratory diseases, 88 (5.3%) from cancer, and 1027 (61.5%) from all other causes. Table 1 shows the baseline characteristics of the participants by quartiles of WCR. The mean (SD) of WCR was 2.71 (0.44). Participants with higher WCR tend to be older, female, unmarried (widowed, divorced, or unmarried), and illiterate. In addition, they were more likely to not smoke or drink, not exercise regularly, have a lower CC and a higher WC, and have an insufficient intake of vegetables.

**Table 1:** Population characteristics by quartiles of waist-calf circumference ratio

Characteristics	Waist-calf circumference ratio				*P* value
	Q1(≤ 2.44)	Q2(2.44—≤ 2.67)	Q3(2.67—≤ 2.93)	Q4(> 2.93)	
No. of participants	1159 (25.0)	1210 (26.2)	1102 (23.8)	1156 (25.0)	
Age (year), mean (SD)	82.12 ± 9.63	82.69 ± 9.52	85.37 ± 9.92	88.70 ± 10.28	< 0.001
Female, no. (%)	472 (40.7)	523 (43.2)	589 (53.4)	836 (72.3)	< 0.001
Married, no. (%)	572 (49.4)	605 (50.0)	450 (40.8)	344 (29.8)	< 0.001
Urban area, no. (%)	484 (41.8)	512 (42.3)	461 (41.8)	491 (42.5)	0.98
Education (year), no. (%)					< 0.001
0	548 (47.3)	591 (48.8)	618 (56.1)	816 (70.6)	
≥ 1	611 (52.7)	619 (51.2)	484 (43.9)	340 (29.4)	
Smoking status, no. (%)					< 0.001
Never	763 (65.8)	790 (65.3)	804 (73.0)	918 (79.4)	
Current	226 (19.5)	248 (20.5)	155 (14.1)	127 (11.0)	
Former	170 (14.7)	172 (14.2)	143 (13.0)	111 (9.6)	
Drinking status, no. (%)					< 0.001
Never	815 (70.3)	846 (69.9)	825 (74.9)	955 (82.6)	
Current	223 (19.2)	219 (18.1)	168 (15.2)	130 (11.2)	
Former	121 (10.4)	145 (12.0)	109 (9.9)	71 (6.1)	
Regular exercise, no. (%)					< 0.001
Never	775 (66.9)	779 (64.4)	778 (70.6)	850 (73.5)	
Current	339 (29.2)	384 (31.7)	296 (26.9)	247 (21.4)	
Former	45 (3.9)	47 (3.9)	28 (2.5)	59 (5.1)	
Intake of fruit, no. (%)					0.99
Never	290 (25.0)	299 (24.7)	272 (24.7)	276 (23.9)	
Occasionally	720 (62.1)	747 (61.7)	687 (62.3)	722 (62.5)	
Almost daily	149 (12.9)	164 (13.6)	143 (13.0)	158 (13.7)	
Intake of vegetables, no. (%)					0.02
Never	25 (2.2)	30 (2.5)	42 (3.8)	45 (3.9)	
Occasionally	467 (40.3)	454 (37.5)	441 (40.0)	479 (41.4)	
Almost daily	667 (57.5)	726 (60.0)	619 (56.2)	632 (54.7)	
Intake of meat, no. (%)					0.11
Never	60 (5.2)	71 (5.9)	87 (7.9)	86 (7.4)	
Occasionally	646 (55.7)	682 (56.4)	608 (55.2)	652 (56.4)	
Almost daily	453 (39.1)	457 (37.8)	407 (36.9)	418 (36.2)	
Intake of fish, no. (%)	190 (16.4)	195 (16.1)	183 (16.6)	223 (19.3)	0.16
Never	190 (16.4)	195 (16.1)	183 (16.6)	223 (19.3)	
Occasionally	897 (77.4)	928 (76.7)	832 (75.5)	842 (72.8)	
Almost daily	72 (6.2)	87 (7.2)	87 (7.9)	91 (7.9)	
Waist circumference (cm), mean (SD)	75.50 ± 9.20	81.30 ± 9.35	83.39 ± 9.82	84.43 ± 9.94	< 0.001
Calf circumference (cm), mean (SD)	34.07 ± 4.34	31.74 ± 3.68	29.84 ± 3.59	25.96 ± 3.97	< 0.001
Body mass index (kg/m^2^), mean (SD)	21.63 ± 3.47	21.91 ± 3.55	22.07 ± 3.66	21.47 ± 3.79	< 0.001
Hypertension, no. (%)	360 (31.1)	409 (33.8)	388 (35.2)	392 (33.9)	0.20
Heart disease, no. (%)	155 (12.3)	161 (13.0)	167 (13.5)	142 (11.4)	0.54
Diabetes mellitus, no. (%)	48 (4.1)	68 (5.6)	66 (6.0)	59 (5.1)	0.22
Cerebrovascular diseases, no. (%)	103 (8.9)	100 (8.3)	85 (7.7)	80 (6.9)	0.35
Respiratory disease, no. (%)	121 (10.4)	154 (12.7)	116 (10.5)	141 (12.2)	0.20
Cancer, no. (%)	10 (0.9)	11 (0.9)	9 (0.8)	9 (0.8)	0.99

Differences in characteristics were compared using the χ^2^ test for categorical variables and ANOVA for continuous variables

*Q* quartile

### Association of anthropometric measures with all-cause mortality

**Fig. 1:**
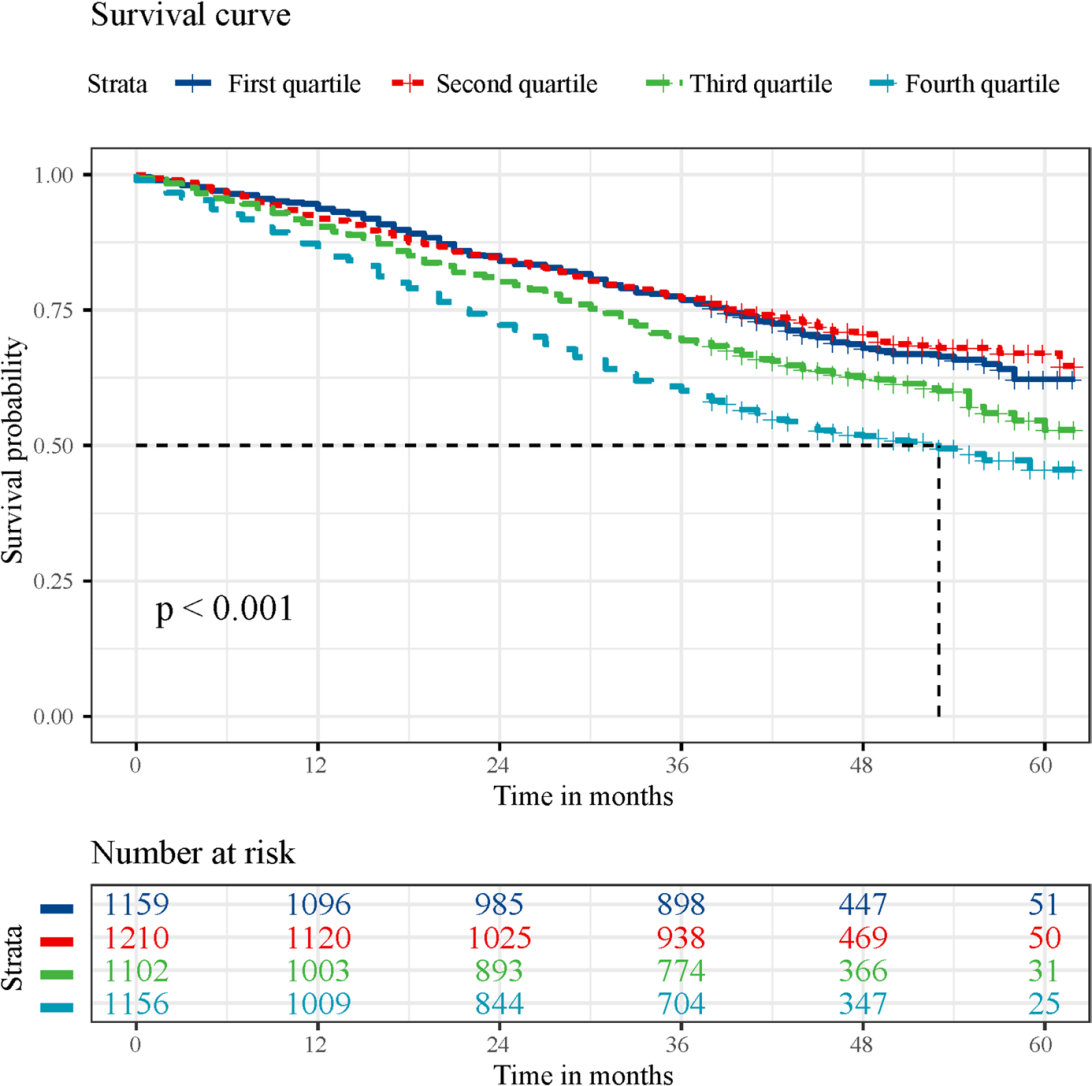
Kaplan–Meier survival curves for all-cause mortality according to waist-calf circumference ratio quartiles. The median survival duration is represented using a vertical dashed line

Based on the Kaplan–Meier curves of survival probability for participants during follow-up, participants with the highest quartile of WCR, the lowest quartile of WC, CC, and BMI at baseline had the lowest survival probabilities compared to those with the remaining quartiles (Fig. 1 and Supplementary Fig. 2–4).

**Fig. 2:**
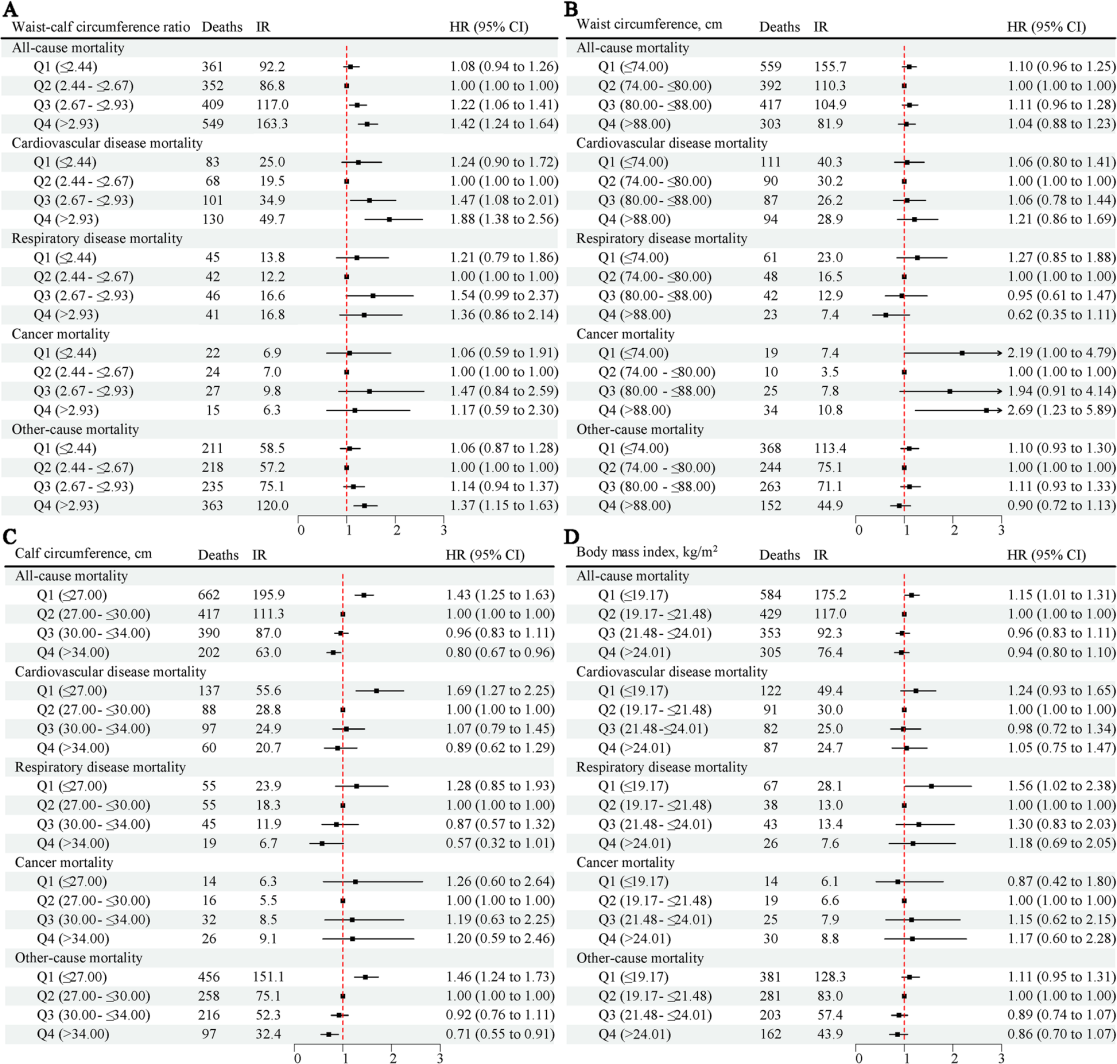
Hazard ratios for all-cause and cause-specific mortality according to anthropometric measures. Multivariate models were adjusted for baseline age, sex, marital status, education, residence, smoking status, drinking status, regular exercise, intake of fruit, intake of vegetables, intake of meat, intake of fish, body mass index, hypertension, heart disease, diabetes mellitus, cerebrovascular disease, respiratory disease, and cancer, and further adjusted for calf circumference in the waist circumference model, waist circumference in the calf circumference model, and waist circumference and calf circumference in the body mass index model. IR, incidence rate (per 1000 person-years); HR, hazard ratio; CI, confidence interval; Q, quartile

After accounting for confounding variables, it was observed that individuals in the fourth and third quartiles of WCR were significantly more likely to experience all-cause mortality, with HRs of 1.42 (95%CI 1.24–1.64) and 1.22 (95% CI 1.06–1.41), respectively, compared to those in the second quartile of WCR (Fig. 2). In contrast, compared to individuals in the second quartile, those in the fourth quartile of CC had a lower risk of all-cause mortality (HR: 0.80, 95% CI: 0.67–0.96), whereas those in the first quartile of CC and BMI had an increased risk of all-cause mortality (HR: 1.43, 95% CI: 1.25–1.63 and HR: 1.15, 95% CI: 1.01–1.31, respectively) (Fig. 2). WC, however, did not show a significant association with all-cause mortality.

**Fig. 3:**
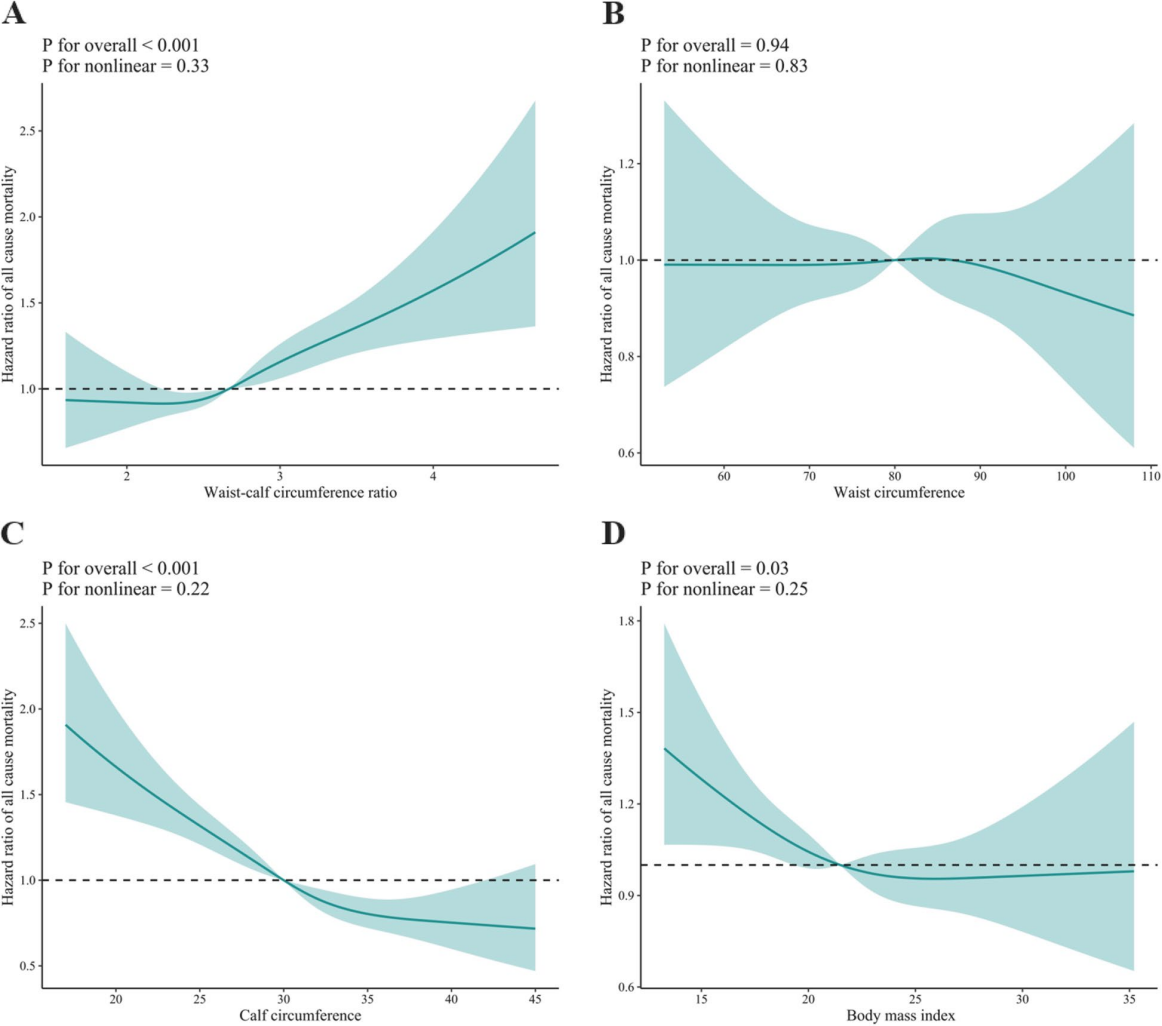
Dose–response association between anthropometric measures and risk of all-cause mortality. Solid red lines are multivariable-adjusted hazard ratios, with shaded areas showing 95% confidence intervals derived from restricted cubic spline regressions with four knots. Multivariate models were adjusted for baseline age, sex, marital status, education, residence, smoking status, drinking status, regular exercise, intake of fruit, intake of vegetables, intake of meat, intake of fish, hypertension, heart disease, diabetes mellitus, cerebrovascular disease, respiratory disease, and cancer, and further adjusted for calf circumference in the waist circumference model, waist circumference in the calf circumference model, and waist circumference and calf circumference in the body mass index model

As shown in Fig. 3, individuals with a high WCR and low CC and BMI had a higher risk of all-cause mortality (*P* for overall < 0.05), while no significant association was found between WC and all-cause mortality. The relationship between WCR and the risk of all-cause mortality followed a linear trend with a J-shaped curve (P for non-linearity = 0.33) (Fig. 3). In contrast, the association between CC and BMI and the risk of all-cause mortality displayed a linear trend with an inverted J-shape (P for non-linearity = 0.22 and 0.25, respectively) (Fig. 3).

### Association of anthropometric measures with cause-specific mortality

The results of the cause-specific mortality analysis are shown in Fig. 2. The fourth quartile of WCR compared to the second quartile was associated with an increased risk of CVD mortality (HR 1.88, 95% CI 1.38–2.56) and other cause mortality (HR 1.37, 95% CI 1.15–1.63). Similarly, the first and fourth quartile of WC compared to the second quartile were associated with cancer mortality, with HRs of 2.19 (95% CI 1.00–4.79) and 2.69 (95% CI 1.23–5.89), respectively. In contrast, the highest CC quartile was associated with a reduced risk of other cause mortality (HR 0.71, 95% CI 0.55–0.91), while the lowest quartile was associated with an increased risk of CVD (HR 1.69, 95% CI 1.27–2.25), and other cause mortality (HR 1.46, 95% CI 1.24–1.73). Additionally, the lowest quartile of BMI was associated with a higher risk of respiratory disease mortality (HR 1.56, 95% CI 1.02–2.38).

**Fig. 4:**
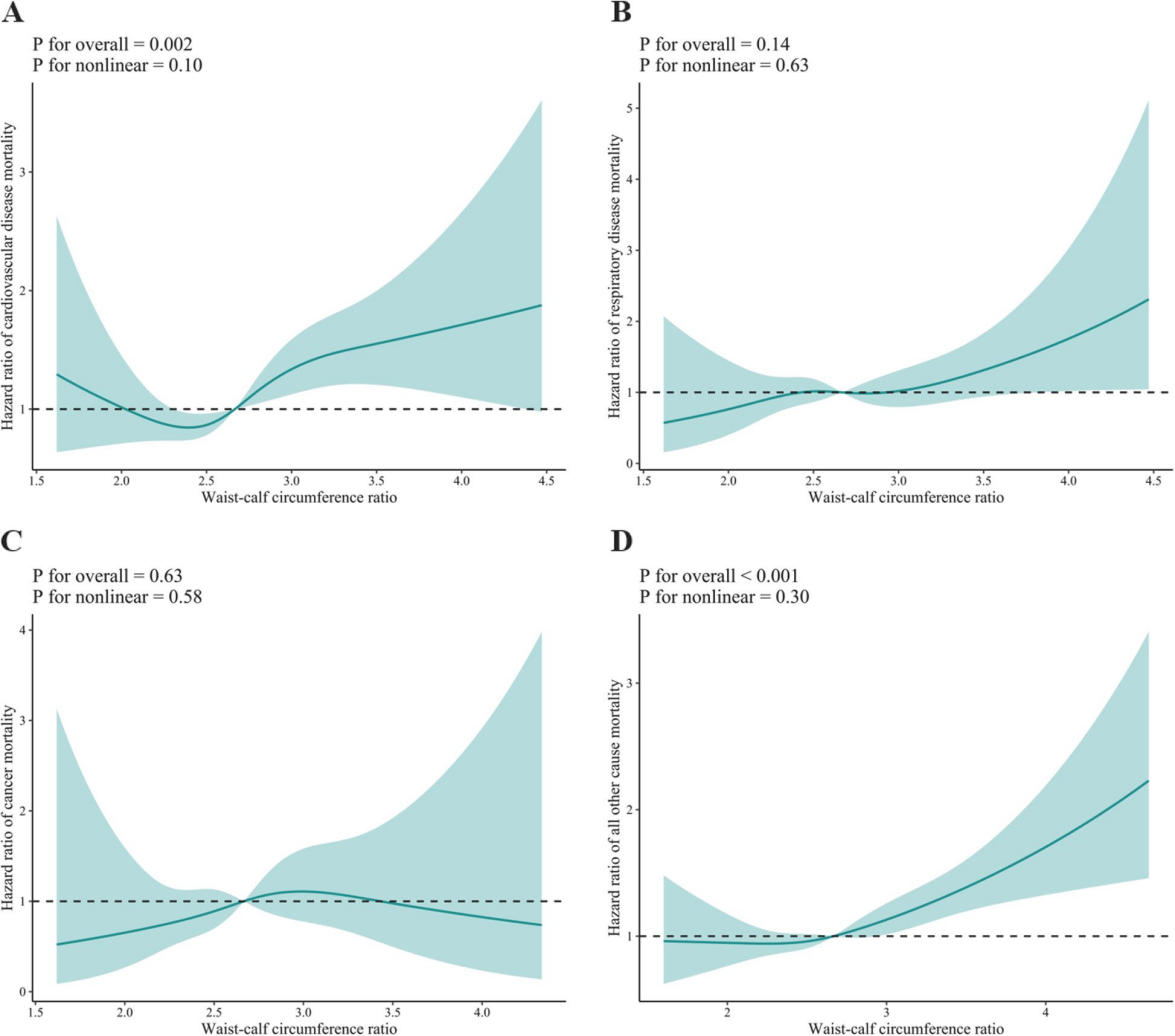
Dose–response association between waist-calf circumference ratio and cause-specific mortality. Solid blue lines are multivariable-adjusted hazard ratios, with shaded areas showing 95% confidence intervals derived from restricted cubic spline regressions with four knots. Multivariate models were adjusted for baseline age, sex, marital status, education, residence, smoking status, drinking status, regular exercise, intake of fruit, intake of vegetables, intake of meat, intake of fish, body mass index, hypertension, heart disease, diabetes mellitus, cerebrovascular disease, respiratory disease, and cancer

Figure 4 illustrates linear trends (J-shaped curves) in both WCR and CVD mortality, as well as other cause mortality (all P for non-linearity > 0.05; all *P* for overall < 0.05). Supplementary Fig. 5 demonstrates non-linear trends in WC and other causes of mortality. Additionally, linear associations (inverse J-shaped curves) were observed between CC and mortality due to CVD and respiratory diseases, as well as other causes of mortality (all P for non-linearity > 0.05; all *P* for overall < 0.05) (Supplementary Fig. 6). Furthermore, the association between BMI and mortality due to other causes was linear, with an inverse J-shaped curve, as shown in Supplementary Fig. 7.

### Subgroup analyses

To further examine the association between anthropometric measures and mortality outcome, we stratified our analysis by the age and sex of the participants. Supplementary Fig. 10 illustrates that the effects of CC on all-cause mortality and CVD mortality were more pronounced in older adults aged ≥ 80 years when compared to those aged < 80 years (P-interaction = 0.006 and 0.045, respectively), while other anthropometric measures and mortality were not significantly modified by age or sex (Supplementary Fig. 8–11).

### Sensitivity analysis

The relationships between anthropometric measures (WCR, WC, CC, and BMI) and mortality from all causes and specific causes remained consistent after excluding 338 participants with missing covariate values (*n* = 4289, Supplementary Fig. 12). Furthermore, when individuals with pre-existing conditions such as heart disease (*n* = 573), diabetes mellitus (*n* = 241), cerebrovascular disease (*n* = 368), and cancer (*n* = 39) were excluded, no substantive changes in the results were observed (*n* = 3624, Supplementary Fig. 13).

## Discussion

This prospective study aimed to investigate the relationship between anthropometric measures and mortality in older adults. Our findings suggest that higher WCR and lower CC were associated with an increased risk of all-cause, CVD, and other-cause mortality. Furthermore, lower BMI was associated with an increased risk of all-cause and respiratory disease mortality, whereas WC was only associated with cancer mortality. WCR may outperform WC or BMI as a predictor of both all-cause and cause-specific mortality. Interaction analyses revealed that the impact of CC on all-cause and CVD mortality was more significant in older adults aged ≥ 80 years. These findings emphasize the importance of considering both central adiposity and muscle mass in mortality risk assessment.

Many studies have reported that lower BMI is associated with excess mortality in older adults [19–21], while others have reported a positive association or no association at all [6, 12]. Our results found that low BMI was associated with a high risk of mortality, particularly respiratory disease mortality, which was in line with previous research [9, 22]. The evidence on the association between BMI and mortality in older adults is conflicting. The controversy surrounding the "obesity paradox" continues. Thus, until conclusive answers are obtained, it is important to consider the potential benefits and drawbacks of weight loss in older adults, including improvements in morbidity and quality of life as well as potential adverse effects**.**

Additionally, numerous studies have investigated the relationship between WC and mortality [6, 20, 23, 24]. However, the findings indicate that the results are inconsistent and diverse. For example, a study of 236 community-dwelling Brazil adults aged ≥ 80 years found that higher WC was associated with reduced all-cause and cardiovascular mortality [6]. Another study of 418 adults aged ≥ 60 years found that a higher WC was associated with an increased risk of CVD mortality but not with all-cause and cancer mortality [24]. However, these studies had some limitations, such as small sample sizes and a lack of information on important confounding factors (dietary intake or smoking). Consistent with our results, the previous study reported a null relation between WC and all-cause mortality in older adults [25]. Interestingly, our study also found that both lower and higher WC have been associated with an increased risk of mortality from cancer. This is likely because low WC can indicate muscle wasting or cachexia, which is a known predictor of poor prognosis. In contrast, high WC is associated with increased levels of visceral fat, which is known to promote insulin resistance and inflammation, both of which can contribute to the development and progression of cancer [26, 27]. However, the relationship between abdominal adiposity and cancer mortality is complex and may depend on the type of cancer and the stage of the disease. The number of deaths from cancer in our sample was relatively small. Therefore, the correlation between WC and cancer needs to be further studied.

Our findings also demonstrated that individuals with a smaller CC exhibited an elevated risk of mortality from all causes, CVD, and other causes. This result aligns with previous studies that have identified CC as a marker of muscle mass [28], which is associated with better physical performance [29] and a lower risk of mortality [30]. However, most prior studies have investigated the relationship between CC and all-cause mortality [31–33]. Only one study that included 418 Brazilian older adults examined the association of CC with cause-specific mortality and found that CC was negatively correlated with all-cause mortality but not with deaths related to CVD or cancer [13]. While these previous studies measured CC directly, the small sample size of the studies limited the generality of their findings. In addition, they also did not assess the effect of central obesity and/or BMI on the results. Our study with a larger sample adds to this literature by demonstrating that CC is a significant predictor of mortality even after controlling for other anthropometric measures (such as BMI and WC) and dietary intake.

Given the opposing impacts of abdominal fat and leg lean mass on disease risk, it may be more relevant to consider the distribution of body fat and lean mass instead of relying solely on BMI and WC to clarify the uncertain associations between obesity and mortality in older adults. Thus, we incorporated the WCR as a metric of the balance between abdominal fat and leg muscle mass. Our findings revealed a noteworthy correlation between an elevated WCR and an increased risk of mortality, encompassing all causes, CVD, and other causes. To our knowledge, no studies have assessed the effects of WCR on mortality at present, thus, comparisons with other studies on the same topic are difficult. The association between WCR and mortality may be explained by several mechanisms. A higher WCR may suggest a greater amount of central adiposity. Increased central adiposity has been linked to insulin resistance [34], oxidative stress [35], and dyslipidemia [36]. Additionally, it can lead to increased production of proinflammatory cytokines, such as interleukin-6 and tumor necrosis factor-alpha [37]. These factors may contribute to the development of chronic diseases, including CVD, and even mortality. Moreover, a higher WCR may also indicate lower muscle mass. Low muscle mass, in turn, is associated with a higher risk of functional decline, disability [29], worse metabolic health [38], and mortality [31]. In contrast, higher muscle mass may be protective against chronic inflammation, which is a common feature of many chronic diseases [39]. Thus, we speculate that the combination of a higher WC and a lower CC, as reflected by a higher WCR, may have an impact on mortality by the cumulative effects of various pathophysiological processes.

It is worth noting that age modified the correlation between CC and mortality. Specifically, the correlation between CC and mortality from all causes and CVD is more pronounced in individuals aged ≥ 80 years compared to those < 80 years. The age difference may be that the oldest adults may be more susceptible to the adverse effects of a low CC due to their reduced physiological reserve and increased vulnerability to stressors [40]. Additionally, vascular health declines with age, and this decline may be more pronounced in individuals over 80 years old. CC is correlated with vascular health measures such as arterial stiffness [41], which may partly explain why it is a stronger predictor of CVD mortality in the oldest adults. The fact that the relationship between CC and mortality risk varies with age underscores the importance of considering age when interpreting health indicators and designing research studies, particularly relevant in the context of an aging population.

### Strengths and limitations

The strengths of this study include its large sample size and prospective design. Trained staff measured WC, CC, height, and weight instead of self-reporting. In addition, the use of a simple and inexpensive measure of central obesity, the WCR, is also a strength of this study. However, several limitations should be considered. First, this study included a relatively short follow-up period, which may not have captured the full extent of the association between anthropometric measures and mortality. Second, the study population consisted of community-dwelling older adults in China, and the results may not be generalizable to other populations. Third, the study was based on a single measurement of anthropometric variables at baseline, which may not reflect changes in body composition over time. Future studies could incorporate longitudinal measurements of anthropometric measures to investigate the association between changes in anthropometric measures and mortality risk in older adults.

## Conclusion

In conclusion, our study suggests that WCR, CC, and BMI are all significant predictors of all-cause mortality in older adults, but the relative importance of these measures varies for cause-specific mortality. WCR and CC may be more useful than WC and BMI in predicting mortality in this population. These findings have important implications for the prevention and management of chronic diseases in older adults. Future studies are needed to confirm and extend our findings, and to explore the mechanisms underlying the association of these measures with mortality. Additionally, comparing the performance of WCR with other measures of body fat distribution, such as visceral adiposity index is also warranted.

### Supplementary Information

**Additional file 1:** **Supplementary Table 1.** The numbers (percentage) of the missing variables. **Supplementary Figure 1.** Flowchart of the included study population. **Supplementary Figure 2.** Kaplan-Meier survival curves for all-cause mortality according to waist circumference quartiles. **Supplementary Figure 3.** Kaplan-Meier survival curves for all-cause mortality according to calf circumference quartiles. **Supplementary Figure 4.** Kaplan-Meier survival curves for all-cause mortality according to body mass index quartiles. **Supplementary Figure 5.** Dose-response association between waist circumference and cause-specific mortality. **Supplementary Figure 6.** Dose-response Association between calf circumference and cause-specific mortality. **Supplementary Figure 7.** Dose-response association between body mass index and cause-specific mortality. **Supplementary Figure 8.** Association of waist-calf circumference ratio with all-cause and cause-specific mortality stratified by age or sex. **Supplementary Figure 9.** Association of waist circumference with all-cause and cause-specific mortality stratified by age or sex. **Supplementary Figure 10.** Association of calf circumference with all-cause and cause-specific mortality stratified by age or sex. **Supplementary Figure 11.** Association of body mass index with all-cause and cause-specific mortality stratified by age or sex. **Supplementary Figure 12.** Sensitivity analyses for the association of anthropometric measures with all-cause and cause-specific mortality after excluding participants with missing covariate data. **Supplementary Figure 13.** Sensitivity analyses for the association of anthropometric measures with all-cause and cause-specific mortality after excluding participants who had diabetes mellitus, heart disease, cerebrovascular disease, and cancer.

## Data Availability

Data are from the Chinese Longitudinal Healthy Longevity Survey, which is a public, open-access repository (https://opendata.pku.edu.cn/dataverse/CHADS).

## Abbreviations

WCR: Waist-calf circumference ratio
WC: Waist circumference
CC: Calf circumference
BMI: Body mass index
HR: Hazard ratio
CI: Confidence interval
CVD: Cardiovascular disease
CLHLS: Chinese Longitudinal Healthy Longevity Survey
SD: Standard deviation
IR: Incidence rate
