# Supplementary material for: Association of waist-calf circumference ratio, waist circumference, calf circumference, and body mass index with all-cause and cause-specific mortality in older adults: a cohort study
Source: BMC Public Health. 2023 Sep 12;23:1777. doi: 10.1186/s12889-023-16711-7 (PMC10498546; doi:10.1186/s12889-023-16711-7)
Supplement: Supplementary file 1 — Additional file 1: Supplementary Table 1. The numbers (percentage) of the missing variables. Supplementary Figure 1. Flowchart of the included study population. Supplementary Figure 2. Kaplan-Meier survival curves for all-cause mortality according to waist circumference quartiles. Supplementary Figure 3. Kaplan-Meier survival curves for all-cause mortality according to calf circumference quartiles. Supplementary Figure 4. Kaplan-Meier survival curves for all-cause mortality according to body mass index quartiles. Supplementary Figure 5. Dose-response association between waist circumference and cause-specific mortality. Supplementary Figure 6. Dose-response Association between calf circumference and cause-specific mortality. Supplementary Figure 7. Dose-response association between body mass index and cause-specific mortality. Supplementary Figure 8. Association of waist-calf circumference ratio with all-cause and cause-specific mortality stratified by age or sex. Supplementary Figure 9. Association of waist circumference with all-cause and cause-specific mortality stratified by age or sex. Supplementary Figure 10. Association of calf circumference with all-cause and cause-specific mortality stratified by age or sex. Supplementary Figure 11. Association of body mass index with all-cause and cause-specific mortality stratified by age or sex. Supplementary Figure 12. Sensitivity analyses for the association of anthropometric measures with all-cause and cause-specific mortality after excluding participants with missing covariate data. Supplementary Figure 13. Sensitivity analyses for the association of anthropometric measures with all-cause and cause-specific mortality after excluding participants who had diabetes mellitus, heart disease, cerebrovascular disease, and cancer. [file 12889_2023_16711_MOESM1_ESM.docx]

**Supplementary Material**

**Supplementary Table 1** The numbers (percentage) of the missing variables.

**Supplementary Figure 1** Flowchart of the included study population.

**Supplementary Figure 2** Kaplan-Meier survival curves for all-cause mortality according to waist circumference quartiles.

**Supplementary Figure 3** Kaplan-Meier survival curves for all-cause mortality according to calf circumference quartiles.

**Supplementary Figure 4** Kaplan-Meier survival curves for all-cause mortality according to body mass index quartiles.

**Supplementary Figure 5** Dose-response association between waist circumference and cause-specific mortality.

**Supplementary Figure 6** Dose-response Association between calf circumference and cause-specific mortality.

**Supplementary Figure 7** Dose-response association between body mass index and cause-specific mortality.

**Supplementary Figure 8** Association of waist-calf circumference ratio with all-cause and cause-specific mortality stratified by age or sex.

**Supplementary Figure 9** Association of waist circumference with all-cause and cause-specific mortality stratified by age or sex.

**Supplementary Figure 10** Association of calf circumference with all-cause and cause-specific mortality stratified by age or sex.

**Supplementary Figure 11** Association of body mass index with all-cause and cause-specific mortality stratified by age or sex.

**Supplementary Figure 12** Sensitivity analyses for the association of anthropometric measures with all-cause and cause-specific mortality after excluding participants with missing covariate data.

**Supplementary Figure 13** Sensitivity analyses for the association of anthropometric measures with all-cause and cause-specific mortality after excluding participants who had diabetes mellitus, heart disease, cerebrovascular disease, and cancer.

**Supplementary Table 1**

The Numbers (percentage) of the missing variables

| Characteristics | Number (%) with missing data |
| --- | --- |
| Marital status | 52 (1.2) |
| Education | 35 (0.8) |
| Smoking status | 11 (0.3) |
| Drinking status | 21 (0.5) |
| Regular exercise | 50 (1.2) |
| Hypertension | 90 (2.1) |
| Heart disease | 99 (2.3) |
| Diabetes mellitus | 106 (2.5) |
| Cerebrovascular disease | 107 (2.5) |
| Respiratory disease | 104 (2.4) |
| Cancer | 125 (2.9) |
| Intake of fruit | 10 (0.2) |
| Intake of vegetable | 10 (0.2) |
| Intake of meat | 27 (0.6) |
| Intake of fish | 28 (0.7) |

Notes: List only the variables with missing data.


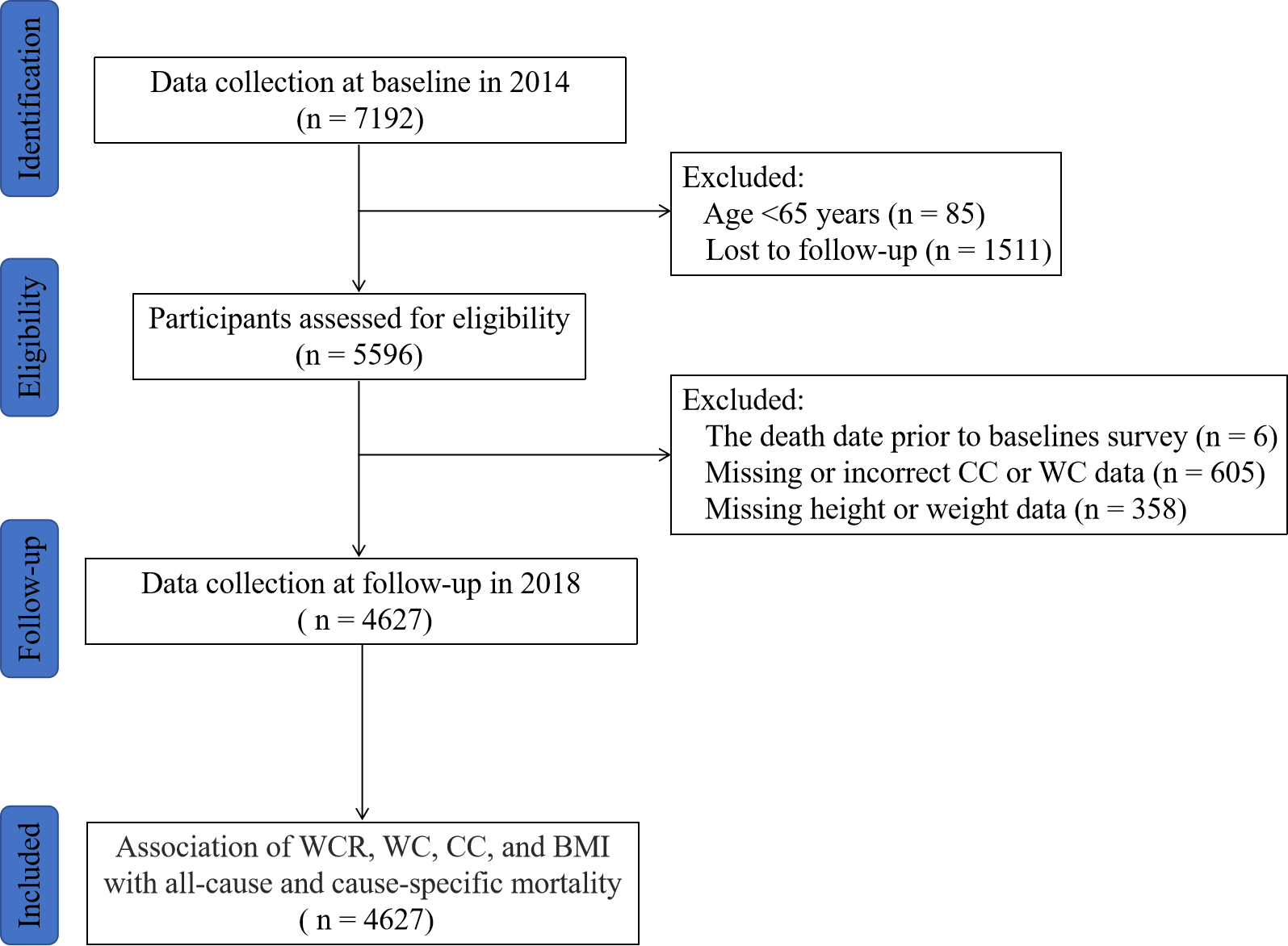


**Supplementary Figure 1** Flowchart of the included study population.

*CC* calf circumference, *WC* waist circumference, *WCR* waist-calf circumference ratio, *BMI* body mass index.


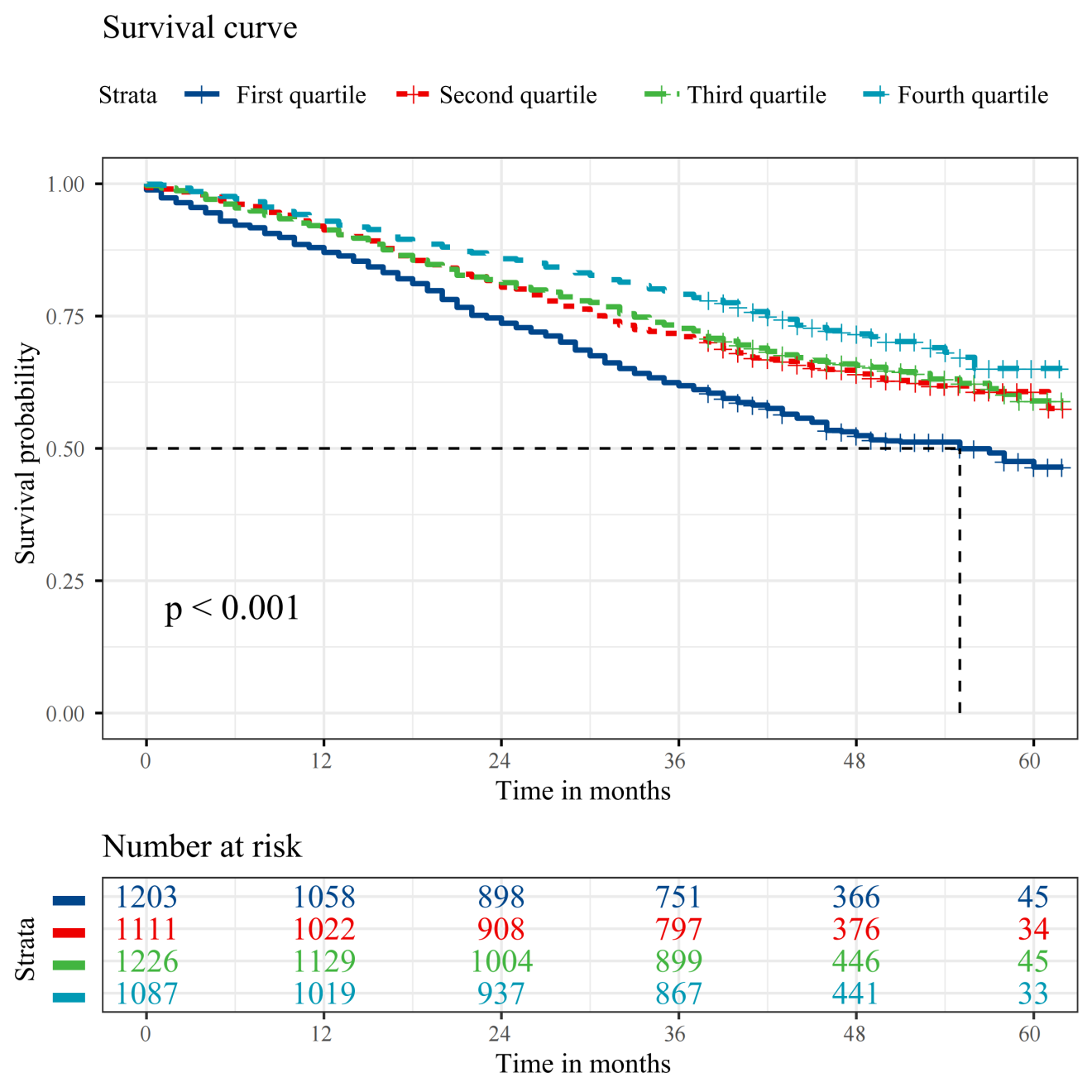


**Supplementary Figure 2** Kaplan-Meier survival curves for all-cause mortality according to waist circumference quartiles.

Notes: The median survival duration is represented using a vertical dashed line.


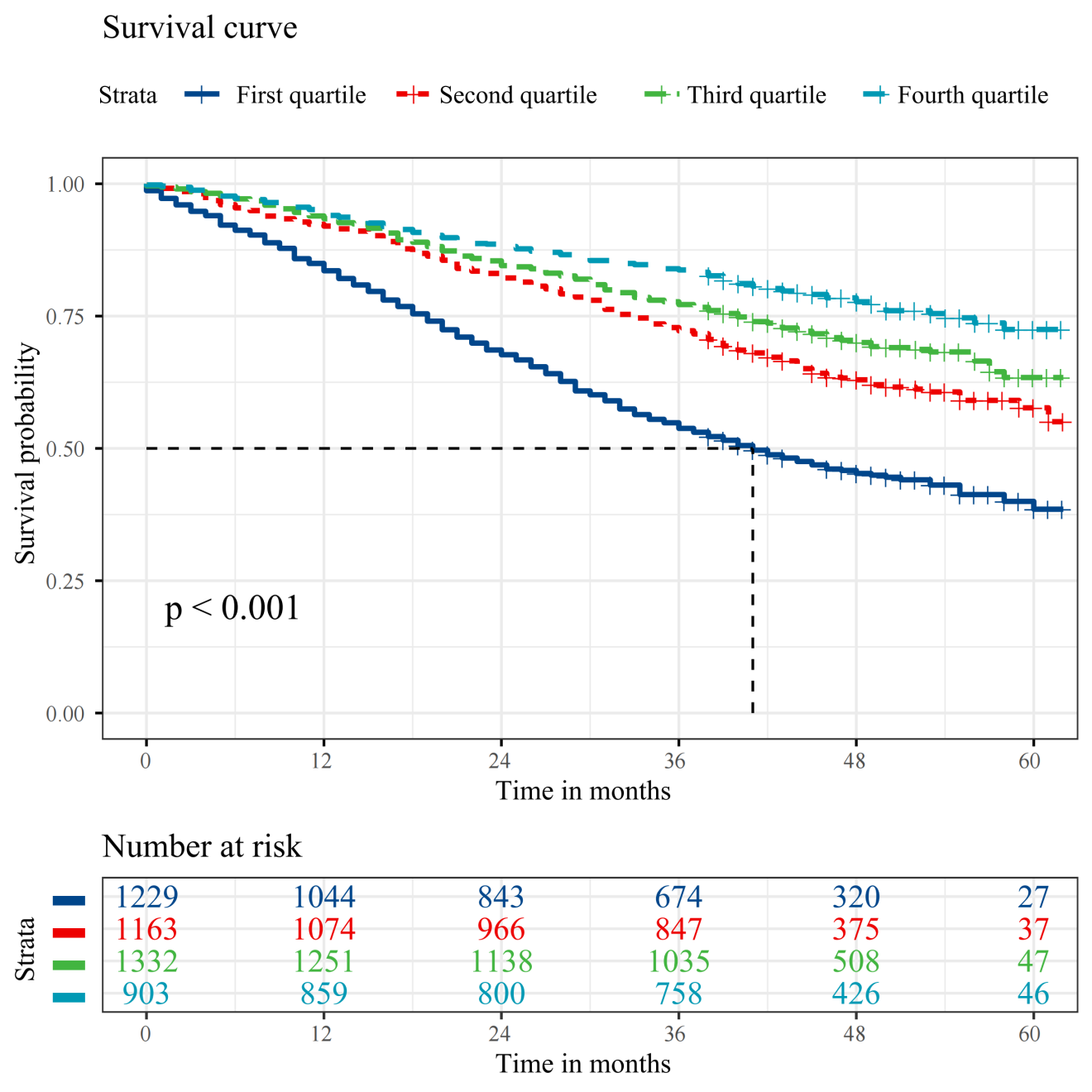


**Supplementary Figure 3** Kaplan-Meier survival curves for all-cause mortality according to calf circumference quartiles.

Notes: The median survival duration is represented using a vertical dashed line.


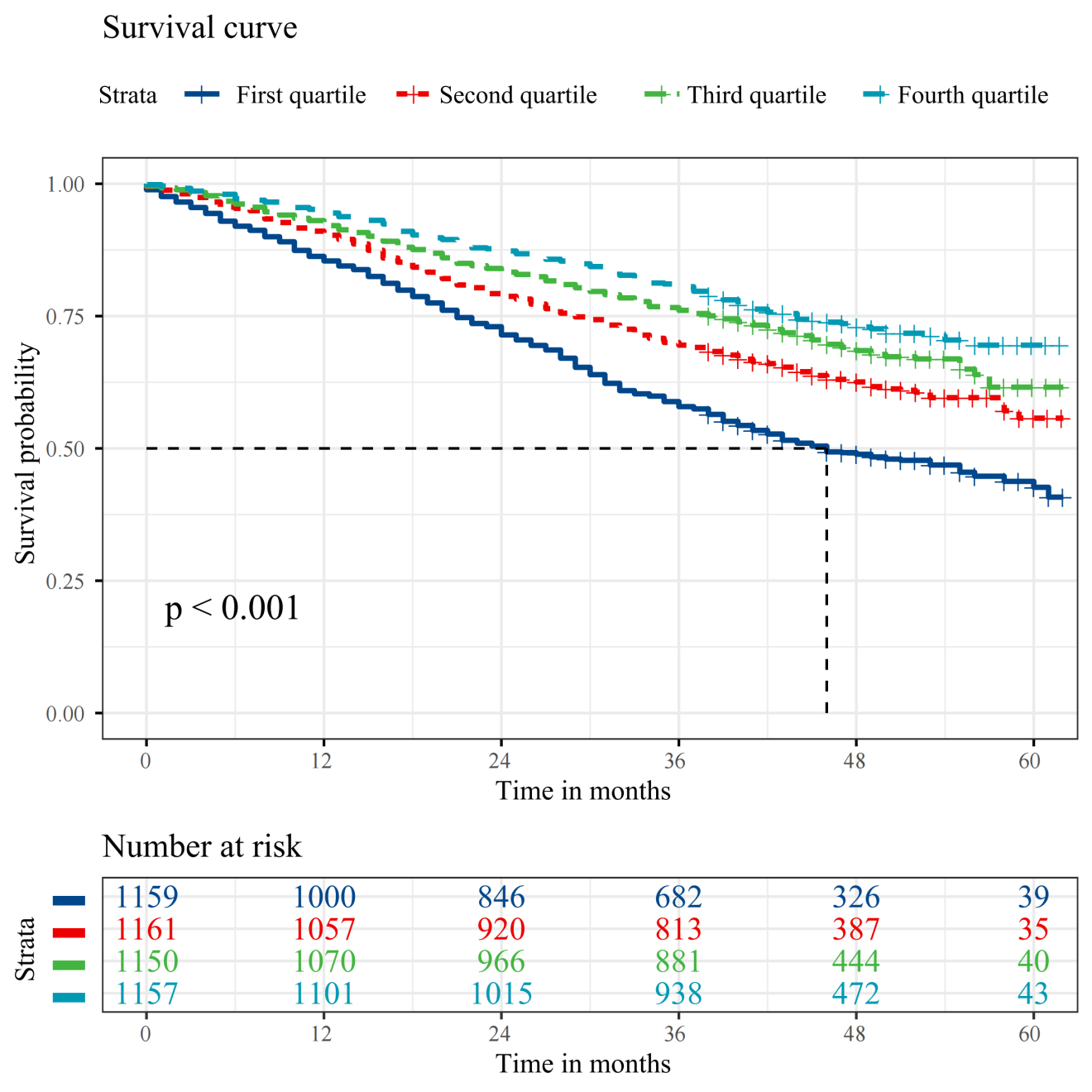


**Supplementary Figure 4** Kaplan-Meier survival curves for all-cause mortality according to body mass index quartiles.

Notes: The median survival duration is represented using a vertical dashed line.


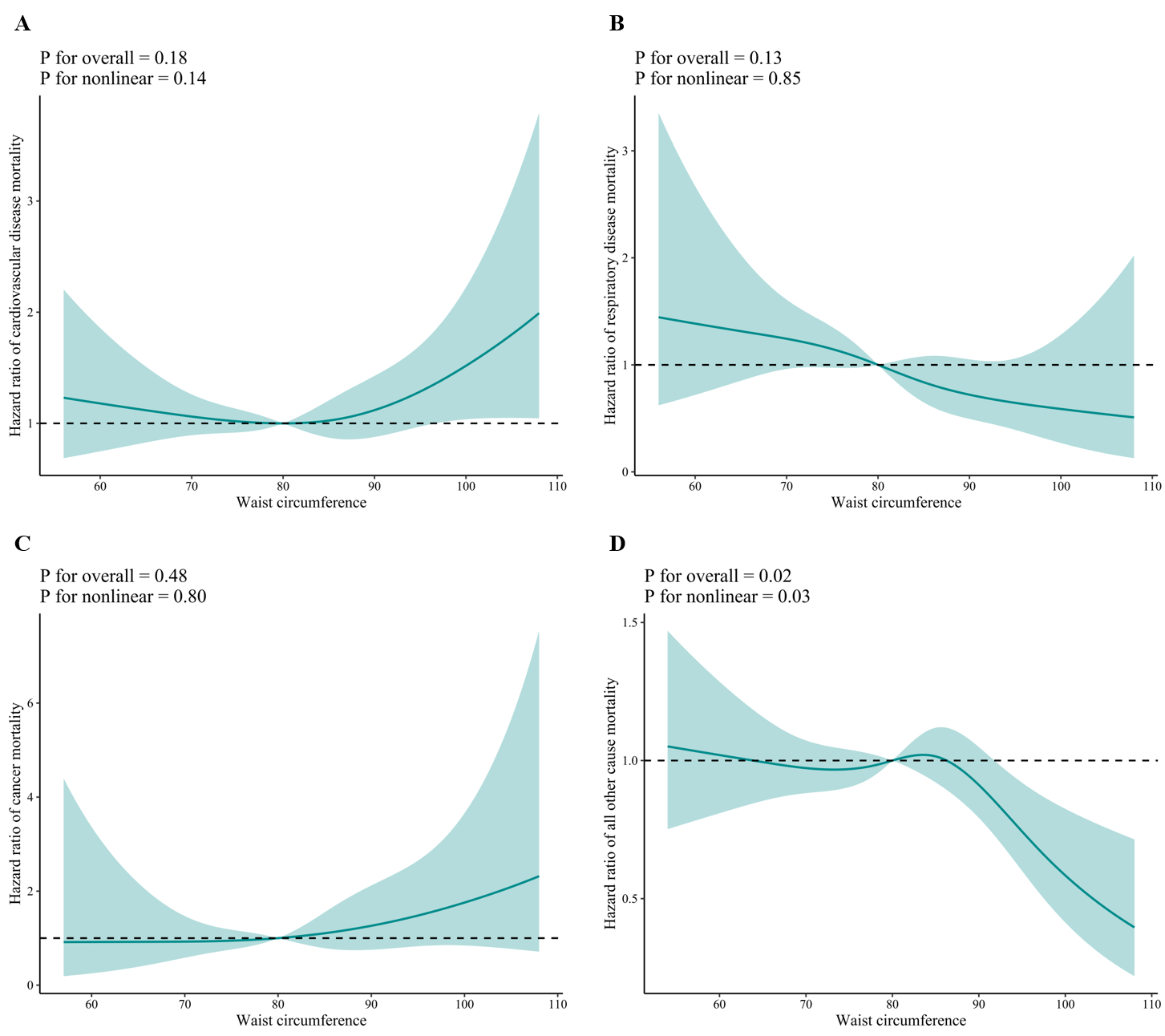


**Supplementary Figure 5** Dose-response association between waist circumference and cause-specific mortality.

Notes: Solid blue lines are multivariable-adjusted hazard ratios, with shaded areas showing 95% confidence intervals derived from restricted cubic spline regressions with four knots. Multivariate models were adjusted for baseline age, sex, marital status, education, residence, smoking status, drinking status, regular exercise, intake of fruit, intake of vegetables, intake of meat, intake of fish, body mass index, calf circumference, hypertension, heart disease, diabetes mellitus, cerebrovascular disease, respiratory disease, and cancer.


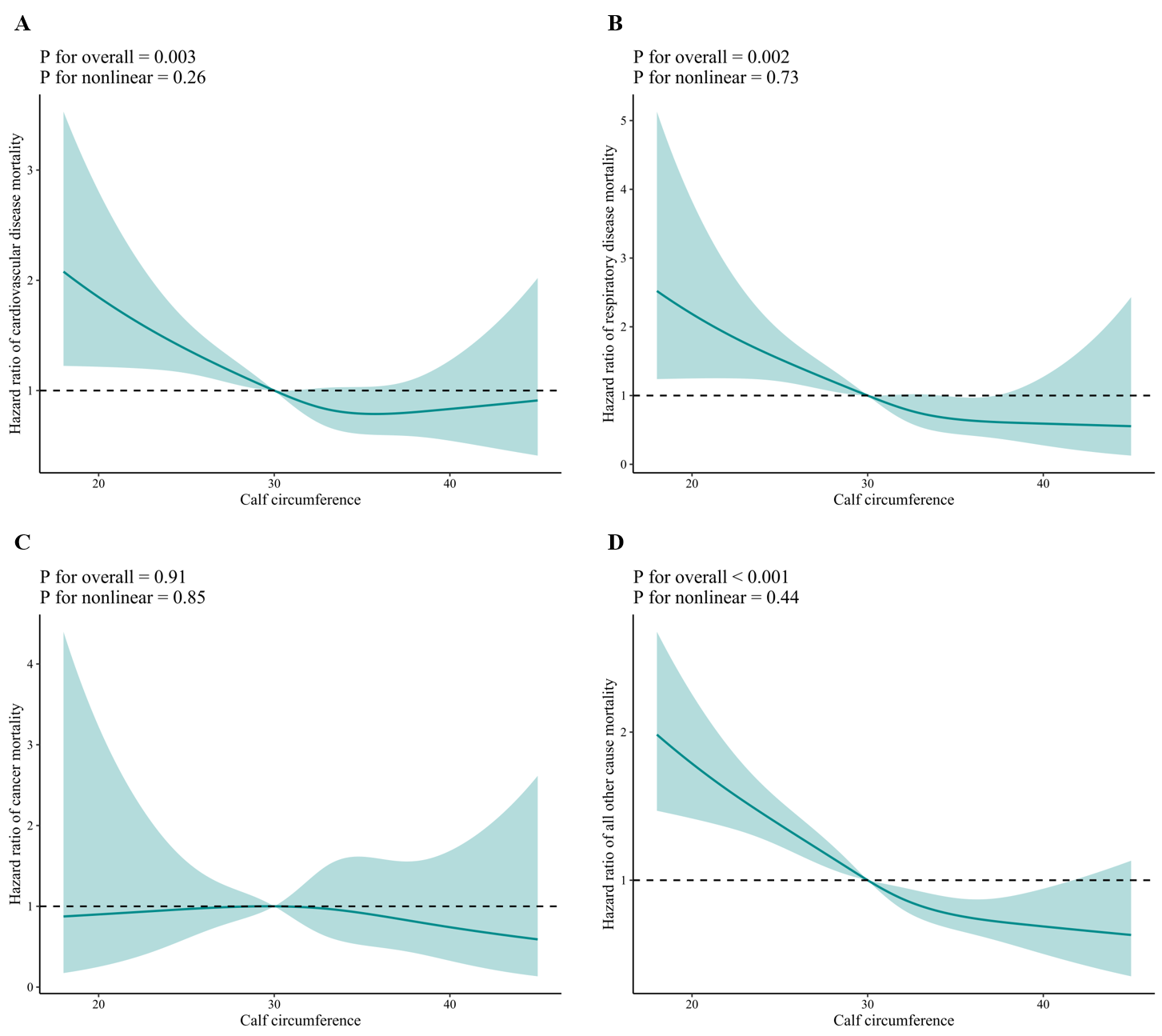


**Supplementary Figure 6** Dose-response Association between calf circumference and cause-specific mortality.

Notes: Solid blue lines are multivariable-adjusted hazard ratios, with shaded areas showing 95% confidence intervals derived from restricted cubic spline regressions with four knots. Reference lines for no association are indicated by dashed black bold lines at a hazard ratio of 1.0. Multivariate models were adjusted for baseline age, sex, marital status, education, residence, smoking status, drinking status, regular exercise, intake of fruit, intake of vegetables, intake of meat, intake of fish, body mass index, waist circumference, hypertension, heart disease, diabetes mellitus, cerebrovascular disease, respiratory disease, and cancer.


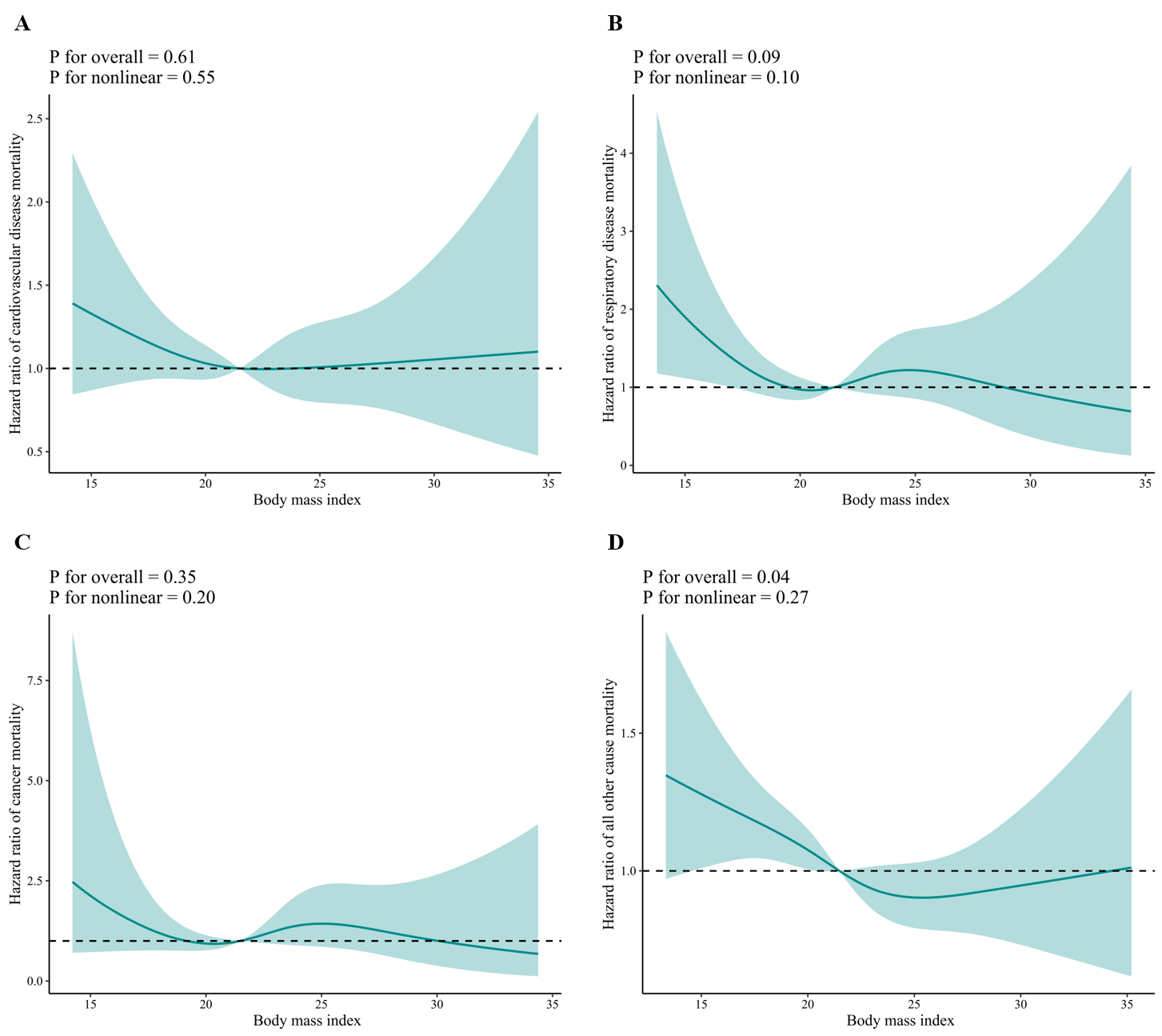


**Supplementary Figure 7** Dose-response association between body mass index and cause-specific mortality.

Notes: Solid blue lines are multivariable-adjusted hazard ratios, with shaded areas showing 95% confidence intervals derived from restricted cubic spline regressions with four knots. Reference lines for no association are indicated by dashed black bold lines at a hazard ratio of 1.0. Multivariate models were adjusted for baseline age, sex, marital status, education, residence, smoking status, drinking status, regular exercise, intake of fruit, intake of vegetables, intake of meat, intake of fish, waist circumference, calf circumference, hypertension, heart disease, diabetes mellitus, cerebrovascular disease, respiratory disease, and cancer.


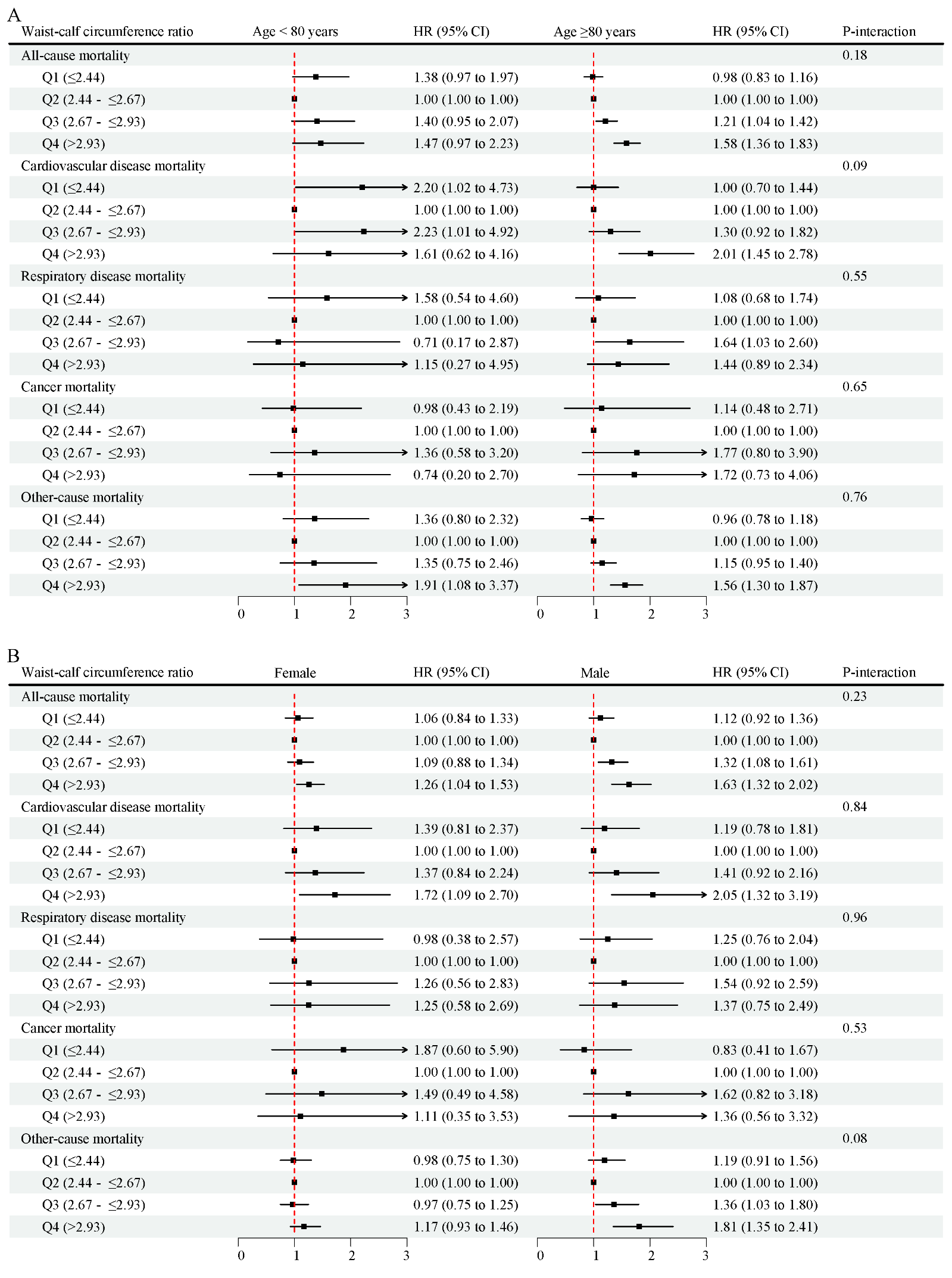


**Supplementary Figure 8** Association of waist-calf circumference ratio with all-cause and cause-specific mortality stratified by age or sex.

*HR* hazard ratio; *CI* confidence interval; *Q* quartile.

Notes: Multivariate models were adjusted for age, sex, marital status, education, residence, smoking status, drinking status, regular exercise, intake of fruit, intake of vegetables, intake of meat, intake of fish, body mass index, hypertension, heart disease, diabetes mellitus, cerebrovascular disease, respiratory disease, and cancer except the stratification factor itself.

**
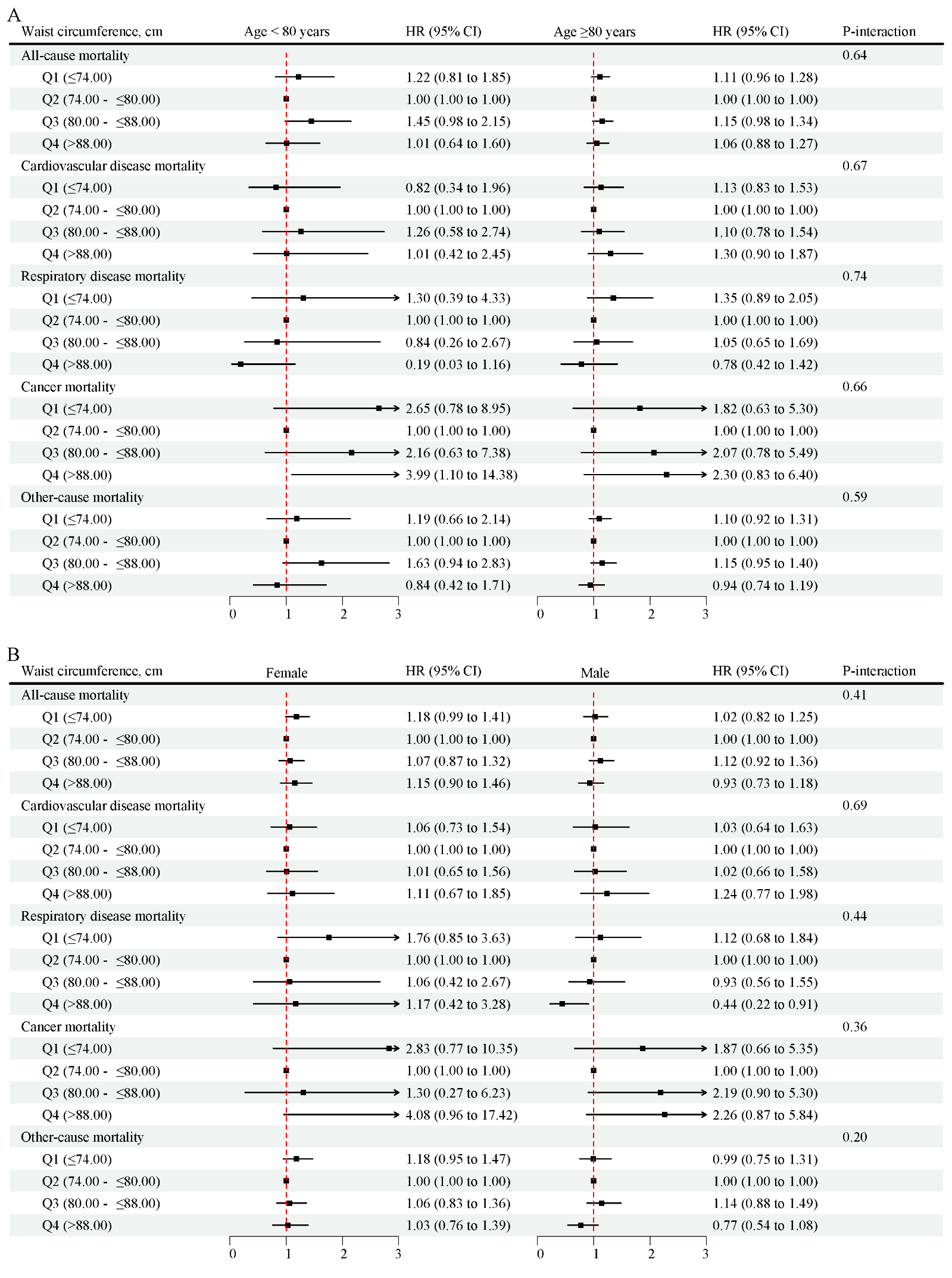
Supplementary Figure 9** Association of waist circumference with all-cause and cause-specific mortality stratified by age or sex.

*HR* hazard ratio; *CI* confidence interval; *Q* quartile.

Notes: Multivariate models were adjusted for age, sex, marital status, education, residence, smoking status, drinking status, regular exercise, intake of fruit, intake of vegetables, intake of meat, intake of fish, body mass index, calf circumference, hypertension, heart disease, diabetes mellitus, cerebrovascular disease, respiratory disease, and cancer except the stratification factor itself.

**
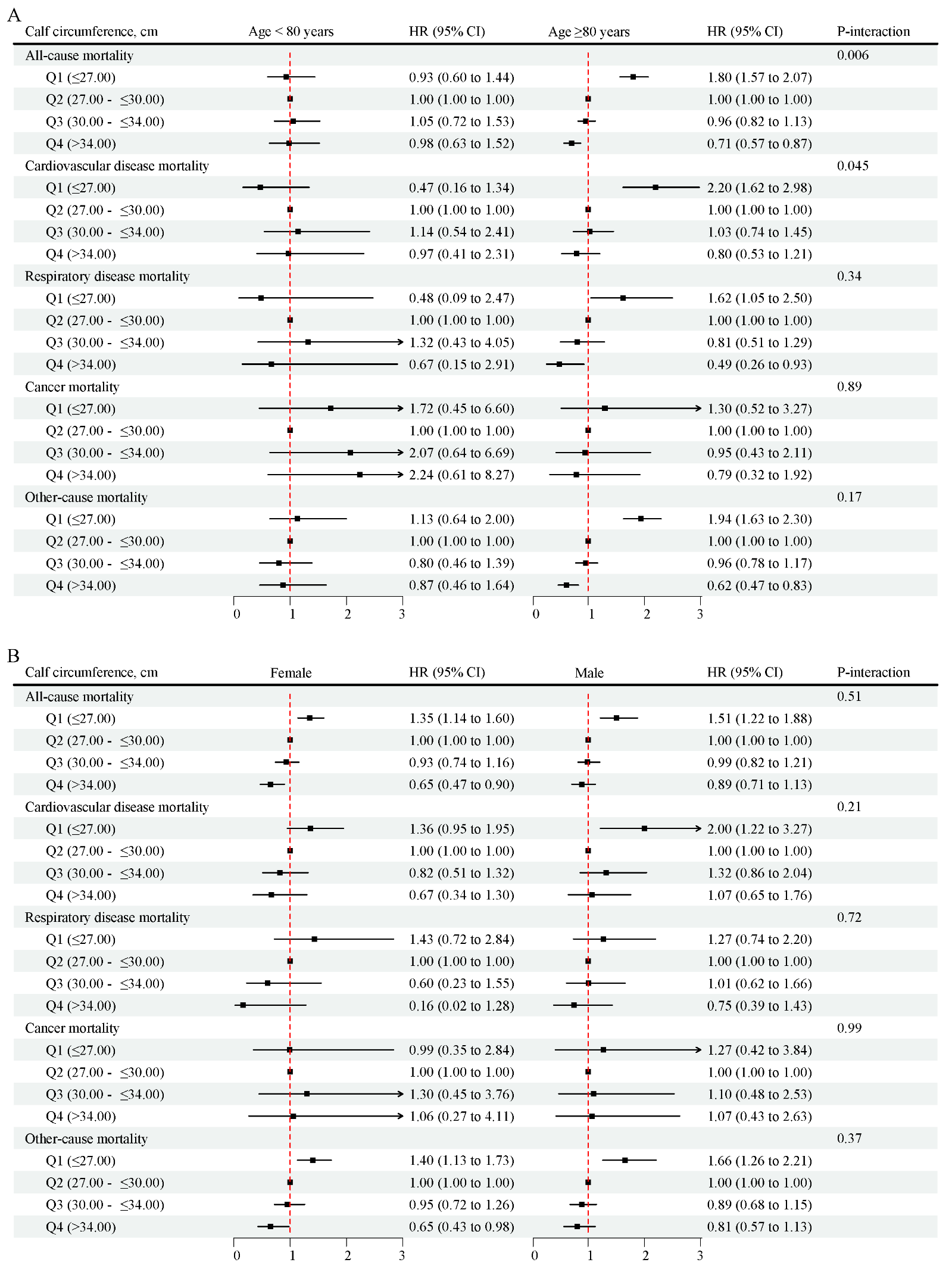
Supplementary Figure 10** Association of calf circumference with all-cause and cause-specific mortality stratified by age or sex.

*HR* hazard ratio; *CI* confidence interval; *Q* quartile.

Notes: Multivariate models were adjusted for age, sex, marital status, education, residence, smoking status, drinking status, regular exercise, intake of fruit, intake of vegetables, intake of meat, intake of fish, body mass index, waist circumference, hypertension, heart disease, diabetes mellitus, cerebrovascular disease, respiratory disease, and cancer except the stratification factor itself.

**
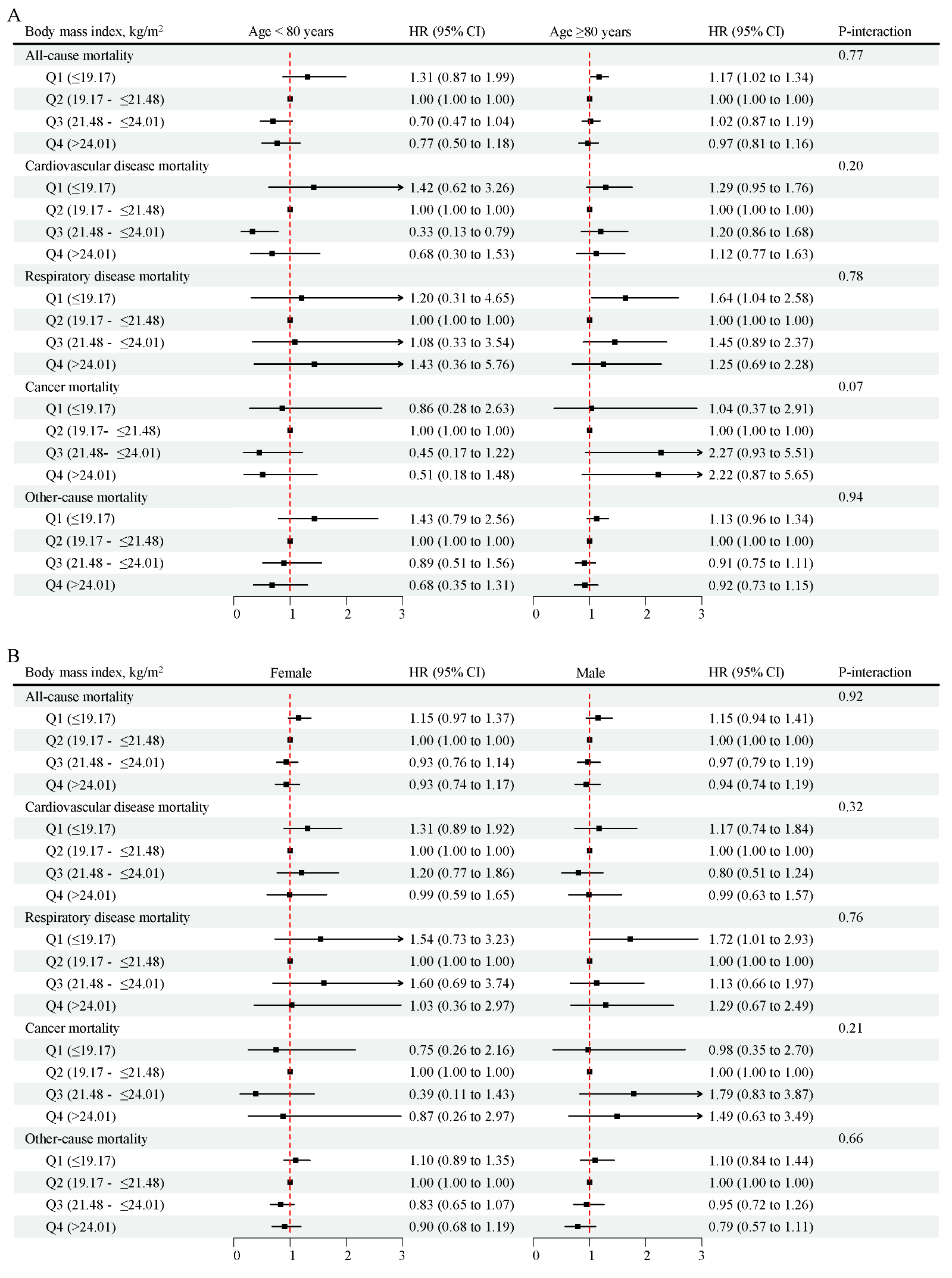
Supplementary Figure 11** Association of body mass index with all-cause and cause-specific mortality stratified by age or sex.

*HR* hazard ratio; *CI* confidence interval; *Q* quartile.

Notes: Multivariate models were adjusted for age, sex, marital status, education, residence, smoking status, drinking status, regular exercise, intake of fruit, intake of vegetables, intake of meat, intake of fish, waist circumference, calf circumference, hypertension, heart disease, diabetes mellitus, cerebrovascular disease, respiratory disease, and cancer except the stratification factor itself.


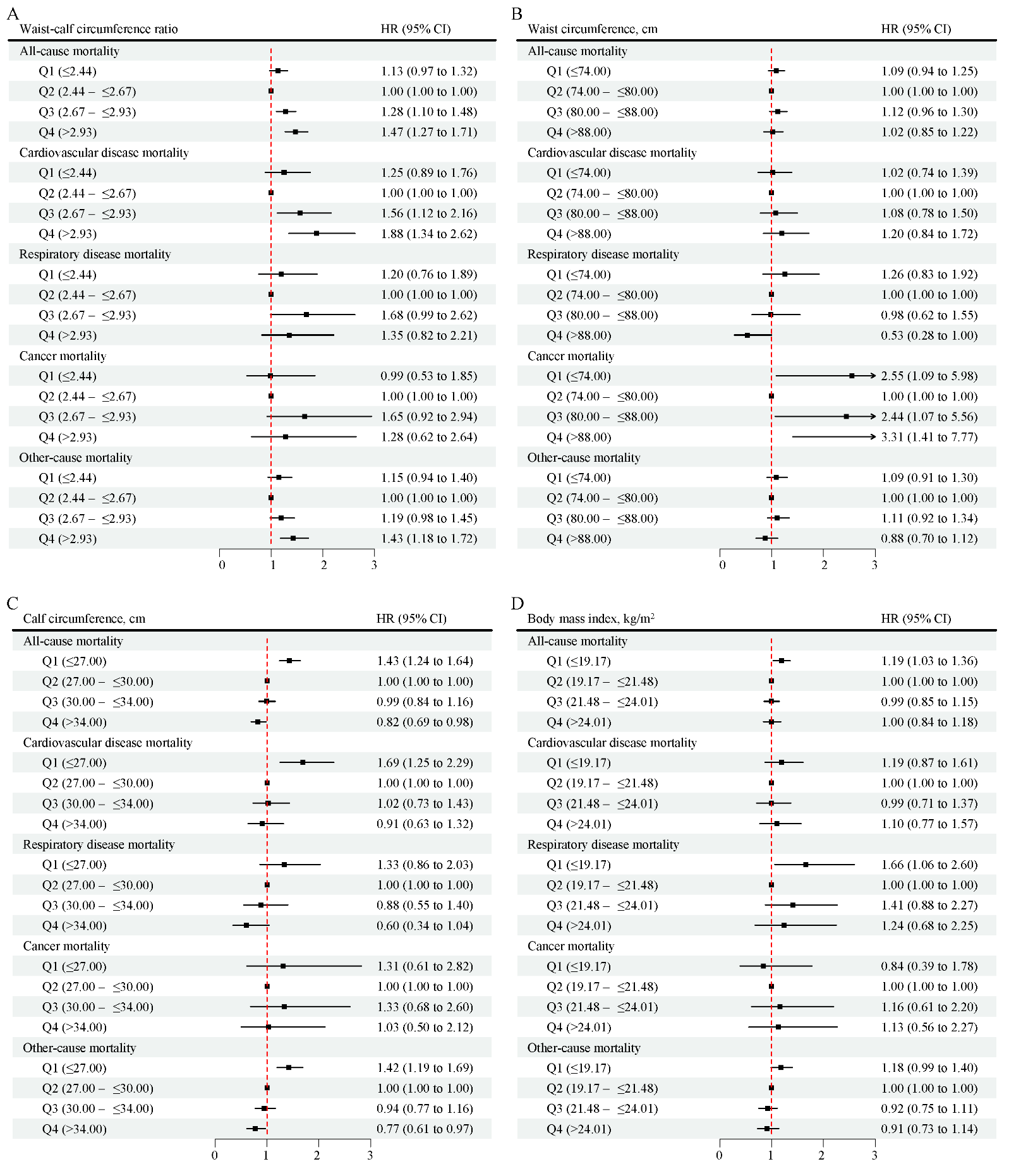


**Supplementary Figure 12** Sensitivity analyses for the association of anthropometric measures with all-cause and cause-specific mortality after excluding participants with missing covariate data.

*HR* hazard ratio; *CI* confidence interval; *Q* quartile.

Notes: Multivariate models were adjusted for age, sex, marital status, education, residence, smoking status, drinking status, regular exercise, intake of fruit, intake of vegetables, intake of meat, intake of fish, body mass index, hypertension, heart disease, diabetes mellitus, cerebrovascular disease, respiratory disease, and cancer, and further adjusted for calf circumference in the waist circumference model, waist circumference in the calf circumference model, and waist circumference and calf circumference in the body mass index model.


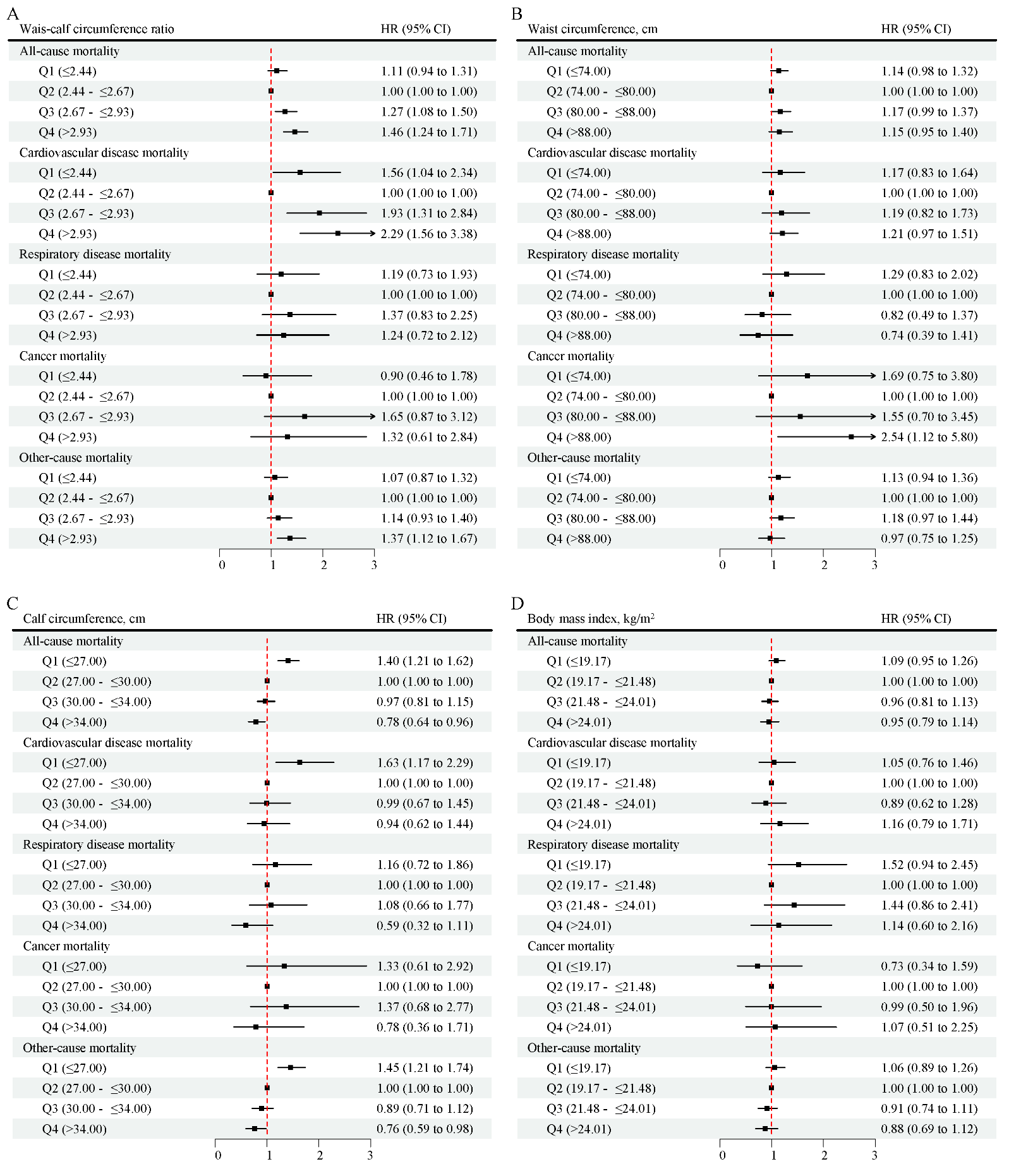


**Supplementary Figure 13** Sensitivity analyses for the association of anthropometric measures with all-cause and cause-specific mortality after excluding participants who had diabetes mellitus, heart disease, cerebrovascular disease, and cancer.

*HR* hazard ratio; *CI* confidence interval; *Q* quartile.

Notes: Multivariate models were adjusted for age, sex, marital status, education, residence, smoking status, drinking status, regular exercise, intake of fruit, intake of vegetables, intake of meat, intake of fish, body mass index, hypertension, and respiratory disease, and further adjusted for calf circumference in the waist circumference model, waist circumference in the calf circumference model, and waist circumference and calf circumference in the body mass index model.
